# Musical Mindset: Within‐Person Links among Social Wellbeing, Depressive Symptoms and Cognition for Persons with Alzheimer's Disease

**DOI:** 10.1002/alz70858_105254

**Published:** 2025-12-26

**Authors:** Samantha Gwendolyn Coleborn, Cynthia McDowell, Nicholas Tamburri, Debra J Sheets, Jodie Gawryluk, Stuart MacDonald

**Affiliations:** ^1^ University of Victoria, Victoria, BC, Canada

## Abstract

**Background:**

Non‐pharmacological lifestyle modifications, including arts‐ and music‐based interventions, have known psychological and cognitive benefits for persons with Alzheimer's Disease and related dementias (ADRD). Although such interventions yield positive results for participants overall, intervention outcomes can vary due to significant individual differences in symptoms (e.g., loneliness, depressive affect) and disease progression. Voices in Motion (ViM) is a longitudinal, intensive repeated‐measures intervention utilizing choral singing to promote psychological and cognitive health for persons with ADRD. The current study explores whether participating in ViM results in systematic time‐varying fluctuations between social wellbeing, depressive symptomology, and cognitive function. We hypothesized that, on occasions when a participant's level of social wellbeing or depressive symptoms were lower relative to their personal average, there would be a corresponding improvement in their cognitive performance. Notably, the novel within‐person design of this study allowed for a more rigorous test of these associations, while controlling for known population‐mean confounds (e.g., age, sex).

**Method:**

Individuals with ADRD (*n* = 32, M age=79.09, SD=7.95, range=57‐98, female=53%) participated in the ViM study. ViM's intensive repeated‐measure burst design included up to 4 assessments within each 3.5‐month choral season, permitting distinct evaluation of between‐ vs. within‐person variance and the use of person‐mean centering, whereby participants served as their own controls. Measures of cognitive function included the Clock Drawing Test, Controlled Oral Word Association Test, and Mini‐Mental State Examination (MMSE). The Patient Health Questionnaire indexed depressive symptomology and the Resources for Enhancing Alzheimer's Caregiver Health II indexed social wellbeing via self‐reported social activity and social support items. Age, education, and sex were included as between‐person covariates for all analyses.

**Result:**

Preliminary multilevel analyses indicated that changes in MMSE scores significantly and positively covaried with social activity satisfaction (*p* < 0.05); on occasions when social activity was higher, cognitive function also improved.

**Conclusion:**

These results suggest social activity satisfaction is associated with within‐person changes in global cognitive functioning for individuals with ADRD, highlighting how a music intervention can foster both social wellbeing and cognitive function. Non‐pharmacological interventions and social prescription hold potential for improving psychological and cognitive health for those with ADRD.

